# ABO and Rhesus blood groups and multiple health outcomes: an umbrella review of systematic reviews with meta-analyses of observational studies

**DOI:** 10.1186/s12916-024-03423-x

**Published:** 2024-05-20

**Authors:** Fang-Hua Liu, Jia-Kai Guo, Wei-Yi Xing, Xue-Li Bai, Yu-Jiao Chang, Zhao Lu, Miao Yang, Ying Yang, Wen-Jing Li, Xian-Xian Jia, Tao Zhang, Jing Yang, Jun-Tong Chen, Song Gao, Lang Wu, De-Yu Zhang, Chuan Liu, Ting-Ting Gong, Qi-Jun Wu

**Affiliations:** 1 Department of Clinical Epidemiology, Shengjing Hospital of China Medical University, Shenyang, China; 2 Clinical Research Center, Shengjing Hospital of China Medical University, Shenyang, China; 3 Hospital Management Office, Shengjing Hospital of China Medical University, Shenyang, China; 4 Department of Obstetrics and Gynecology, The Fourth Affiliated Hospital of China Medical University, Shenyang, China; 5 Department of Radiology, Shengjing Hospital of China Medical University, Shenyang, China; 6 Department of Pediatrics, Shengjing Hospital of China Medical University, Shenyang, China; 7 Department of Hematology, Shengjing Hospital of China Medical University, Shenyang, China; 8 Department of Otolaryngology Head and Neck Surgery, Shengjing Hospital of China Medical University, Shenyang, China; 9 Department of Endocrinology, Shengjing Hospital of China Medical University, Shenyang, China; 10 School of Medicine, Zhejiang University, Hangzhou, China; 11 Cancer Epidemiology Division, Population Sciences in the Pacific Program, University of Hawaii Cancer Center, University of Hawaii at Manoa, Honolulu, HI USA; 12 NHC Key Laboratory of Advanced Reproductive Medicine and Fertility (China Medical University), National Health Commission, Shenyang, China

**Keywords:** ABO blood group, Meta-analysis, Observational study, Rhesus blood group, Umbrella review

## Abstract

**Background:**

Numerous studies have been conducted to investigate the relationship between ABO and Rhesus (Rh) blood groups and various health outcomes. However, a comprehensive evaluation of the robustness of these associations is still lacking.

**Methods:**

We searched PubMed, Web of Science, Embase, Scopus, Cochrane, and several regional databases from their inception until Feb 16, 2024, with the aim of identifying systematic reviews with meta-analyses of observational studies exploring associations between ABO and Rh blood groups and diverse health outcomes. For each association, we calculated the summary effect sizes, corresponding 95% confidence intervals, 95% prediction interval, heterogeneity, small-study effect, and evaluation of excess significance bias. The evidence was evaluated on a grading scale that ranged from convincing (Class I) to weak (Class IV). We assessed the certainty of evidence according to the Grading of Recommendations Assessment, Development, and Evaluation criteria (GRADE). We also evaluated the methodological quality of included studies using the A Measurement Tool to Assess Systematic Reviews (AMSTAR). AMSTAR contains 11 items, which were scored as high (8–11), moderate (4–7), and low (0–3) quality. We have gotten the registration for protocol on the PROSPERO database (CRD42023409547).

**Results:**

The current umbrella review included 51 systematic reviews with meta-analysis articles with 270 associations. We re-calculated each association and found only one convincing evidence (Class I) for an association between blood group B and type 2 diabetes mellitus risk compared with the non-B blood group. It had a summary odds ratio of 1.28 (95% confidence interval: 1.17, 1.40), was supported by 6870 cases with small heterogeneity (*I*^2^ = 13%) and 95% prediction intervals excluding the null value, and without hints of small-study effects (*P* for Egger’s test > 0.10, but the largest study effect was not more conservative than the summary effect size) or excess of significance (*P* < 0.10, but the value of observed less than expected). And the article was demonstrated with high methodological quality using AMSTAR (score = 9). According to AMSTAR, 18, 32, and 11 studies were categorized as high, moderate, and low quality, respectively. Nine statistically significant associations reached moderate quality based on GRADE.

**Conclusions:**

Our findings suggest a potential relationship between ABO and Rh blood groups and adverse health outcomes. Particularly the association between blood group B and type 2 diabetes mellitus risk.

**Supplementary Information:**

The online version contains supplementary material available at 10.1186/s12916-024-03423-x.

## Background

Blood groups can be categorized based on different systems, such as the ABO blood group system, the Rhesus (Rh) blood group system, and the MN blood group system [1]. ABO blood group system is the most frequently applied [2]. Each of the two alleles possesses antigen A, B, or neither. These alleles come together to be a combination, determining an individual’s blood type phenotype, thus perform as the type of O, A, B, or AB. The Rh blood group system is more polymorphic than others among human blood groups, which is composed of numerous antigens and next to ABO. The ABO and Rh blood group system are extensively utilized in clinical practice, affecting host susceptibility [3, 4].

The previous study suggested that blood groups are involved in disease mechanisms at the molecular level mediated either through the blood group antigens or by the blood group reactive antibodies [5]. In addition, J. Höglund et al. found 39 plasma proteins were associated with variation at the ABO locus. For example, proteins with functions related to tumorigenesis (CA9, Gal-9, and KLK6) and pro-inflammatory or anti-inflammatory functions (IFN-gamma-R1, IL-18BP, and MARCO) [6]. Generally, the overexpression of these proteins leads to an abnormal cell proliferation or cell growth. Thus, blood group may influence disease development through protein expression levels.

Numerous systematic reviews with meta-analyses have been published, which explored correlations between ABO and Rh blood groups with various health outcomes [7–9]. However, to date, the association between these blood groups and human health outcomes remains controversial [10–12]. Most of them have primarily concentrated on one single disease end-point, lacking a comprehensive evaluation of the aforementioned relationships. In addition, the strength and reliability of the evidence remains unclear. To overcome the inherent limitations of systematic reviews with meta-analyses and provide a comprehensive overview of the claimed associations of ABO and Rh blood groups with health outcomes, in the form of an umbrella review (UR), is necessary.

UR synthesizes evidence from various systematic reviews with meta-analyses on a subject, appraising the certainty, precision, and potential bias of the correlations, thus facilitating evidence grading based on well-defined criteria [13]. We set out to conduct an UR to comprehensively evaluate systematic reviews with meta-analyses of observational studies, which examined associations of ABO and Rh blood groups with a range of health outcomes. This endeavor was aimed at presenting an overview of the breadth and validity for aforementioned associations. We thus hoped to provide both clinicians and policy makers with robust data to identify high-risk groups and inform clinical practice and guidelines.

## Methods

### Protocol registration

We have gotten the registration for the protocol of this UR with the International Prospective Register of Systematic Reviews (PROSPERO; registration number CRD42023409547). The study followed the Preferred Reporting Items for Systematic Reviews and Meta-analyses reporting guideline [14] (Additional file 1: Table S1) and the Meta-analysis of Observational Studies in Epidemiology reporting guideline [15] (Additional file 1: Table S2).

### Search strategy

We systematically searched PubMed, Web of Science, Embase, Scopus, Cochrane Library, and several regional databases (Latin American and Caribbean Health Sciences Literature, Western Pacific Region Index Medicus, Index Medicus for South-East Asia Region, Index Medicus for the Eastern Mediterranean Region, and African Index Medicus) on the date from inception until Feb 16, 2024, to identify systematic reviews with meta-analyses of observational studies evaluating associations between ABO as well as Rh blood groups and diverse health outcomes. We used the keywords (“ABO” OR “blood group” OR “blood type” OR “Rh”) AND (“meta-analysis” OR “systematic review” OR “systematic overview”) (Additional file 1: Table S3) to search. Besides, the literature search was reviewed by hand-checking the reference lists of all systematic reviews with meta-analyses.

### Eligibility criteria

Articles were selected based on the following PECOS (Population, Exposure, Comparison, Outcome, Study design) strategy:

1. Population: population with ABO or Rh blood groups;
2. Exposure: ABO (blood types A, B, O, and AB) and Rh (Rh positive [Rh +] and Rh negative [Rh −]) blood groups (any method used to assess blood type, including genetic tests and forward/reverse agglutination tests, was accepted);
3. Comparison: different blood groups;
4. Outcome: any health outcome (e.g., cancer, coronavirus disease 2019 [COVID-19], coronary artery disease, etc.). Ascertained health outcomes using self-report, observed (e.g., clinical diagnoses) or objective [e.g., biomarkers, certified mortality] criteria); and
5. Study design: systematic reviews with meta-analyses of observational studies (cohort, case–control, or cross-sectional studies).

The exclusion criteria were established as follows: (1) systematic reviews without quantitative analysis, (2) systematic reviews with meta-analyses without study-level data (e.g., effect sizes, 95% confidence intervals [CIs], the number of cases, and participants/control), (3) studies on genetic polymorphisms, animal studies, laboratory studies, conference abstracts and randomized controlled trials, or (4) systematic reviews with meta-analyses conducted in languages other than English.

Given the requirement for a minimum of three original studies to calculate 95% prediction intervals (PIs), we incorporated meta-analyses comprising at least 3 original studies [16]. Associations were considered to overlap if they assessed the same research topic and were examined in more than one systematic review with meta-analysis [17]. The inclusion of primary studies once or more may be led by incorporating results of reviews with overlapping associations, and biased findings and estimates could be caused by incorporating results as well [18, 19]. Therefore, the systematic review with meta-analysis which contained the largest number of primary studies was picked up if two or more systematic reviews with meta-analyses overlapped, while the one with the largest sample size of participants if more than one systematic review with meta-analysis kept the same numbered primary studies.

To ascertain the eligible articles, four experienced investigators (Y-JC, J-KG, J-TC, and YY) matched in pairs and screened titles, abstracts, and full texts independently. We also checked the references of relevant studies to confirm any other eligible articles by hand. If there were any discrepancies, they would be made out by a third reviewer (Q-JW).

### Data extraction

Ten trained investigators (Y-JC, J-KG, YY, X-XJ, W-JL, T-Z, YY, MY, ZL, and X-LB) were paired to extract data independently, discrepancies were settled by a third reviewer (Q-JW) when it was needed. From every meta-analysis we identified, it was abstracted of the contents on the name of the first author, journal, publication year, exposures of interest, outcomes of interest, comparison, meta-analysis metrics (RR [risk ratio], OR [odds ratio], or HR [hazard ratio]), and the number of studies considered. From the individual studies included in every meta-analysis, it was extracted of the name of the first author, publication year, epidemiological study design, number of cases and controls in the observational case–control studies or total population in the observational cohort studies, maximally adjusted risk estimates, and 95% CIs.

### Data analysis

*Estimation of summary effect*—We utilized a random-effects model for each meta-analysis to do a calculation for the summary effect size and corresponding 95% CI [20].

*Estimation of prediction interval*—We got the 95% prediction intervals (PIs) for the summary random effect sizes, because it can explain heterogeneity between varied studies and the uncertainty for the effect, with an expectation in another study concerning on the same relationship [21].

*Assessment of heterogeneity*—We evaluated heterogeneity with the *I*^2^ metric. And *I*^2^ value exceeding 50% is judged large heterogeneity, and 75% is judged very large heterogeneity similarly [22]. We also produced *τ*^2^ statistic to assess the heterogeneity.

*Assessment of small study effects*—Through Egger’s regression asymmetry test [23], we evaluated small-study effects (i.e., whether larger studies are more likely to give indirectly smaller estimates of effect size when compared with smaller ones) [24]. Reasons for distinctions between small and large studies such as publication and other reporting biases, genuine heterogeneity, chance, or other conditions are revealed through small study effects [24]. They were considered to exist when the largest study effect was more conservative than the summary effect size in the meta-analysis and it was found that *P* value < 0.10 in the regression asymmetry test.

*Evaluation of excess significance*—We assessed excess significance bias by analyzing whether the number of observed studies (*O*) with nominally statistically significant results (“positive” studies, *P* < 0.05) was larger than the expected number of studies (*E*) with statistically significant results using the chi-square test [25]. The effect size of the largest study (that is, the smallest standard error) in a meta-analysis assessed the strength of the study which needed to use a noncentral t distribution [26, 27]. The excess significance test was judged positive when it comes to both *O* > *E* and *P* < 0.10 [22].

*Strength of evidence*—According to the established criteria applied in previously published URs [13, 28–30] and based on our calculation, significant associations (*P* < 0.05) between ABO and Rh blood groups and health outcomes were divided into 4 levels of evidence strength (convincing [Class I], highly suggestive [Class II], suggestive [Class III], or weak [Class IV] evidence) to draw conclusions. This criterion was evaluated based on statistical significance, number of cases, heterogeneity, largest study, 95% PI, small-study effect, and excess significance bias. *P* value ≥ 0.05 demonstrated a statistically non-significant association (Additional file 1: Table S4).

*Certainty of the evidence*—The credibility of the evidence was qualitatively assessed by two reviewers (W-YX and X-LB) using the GRADE (Grading of Recommendations, Assessment, Development, and Evaluations) method. As recommended by GRADE, the level of evidence was graded the high, moderate, low, and very low determined by risk of bias, inconsistency, indirectness, imprecision, and publication bias.

*Sensitivity analyses*—To verify the robustness of our findings, we conducted sensitivity analyses to assess the concordance of the summary associations, which were initially graded as convincing (Class I) or highly suggestive (Class II) evidence. The sensitivity analyses were realized by excluding small-sample studies (< 25th percentile) from meta-analyses with evidence of small-study effects and primary studies with low-quality evidence (Newcastle–Ottawa Scale < 6 [31], Agency for Healthcare Research and Quality < 8 [32], or effective public health practice project guideline rating moderate and low rather than strong quality [33]. Further sensitivity analysis was performed with the meta-analyses due to overlap in the main analysis. All statistical analyses were conducted in STATA version 17 and RStudio version 3.6.2.

### Assessment of the methodological quality of meta-analyses

We used A Measurement Tool to Assess Systematic Reviews (AMSTAR) to evaluate the quality of systematic reviews and meta-analyses, which was considered as a valid and dependable measurement tool [34]. This instrument contains a total of 11 items. A “yes” scores one point, and the other answers score 0 points. The AMSTAR was graded as low (0–3 points), moderate (4–7 points), or high quality (8–11 points) [34]. Ten trained investigators (Y-CS, Z-PN, W-YX, YY, W-JL, ZL, JY, X-LB, MY, and J-NS) matched in pairs, and AMSTAR was used independently to assess the eligible systematic reviews with meta-analyses on methodological quality. Disagreements were made the final decision by the third author (Q-JW).

## Results

### Literature identification and selection

**Fig. 1:**
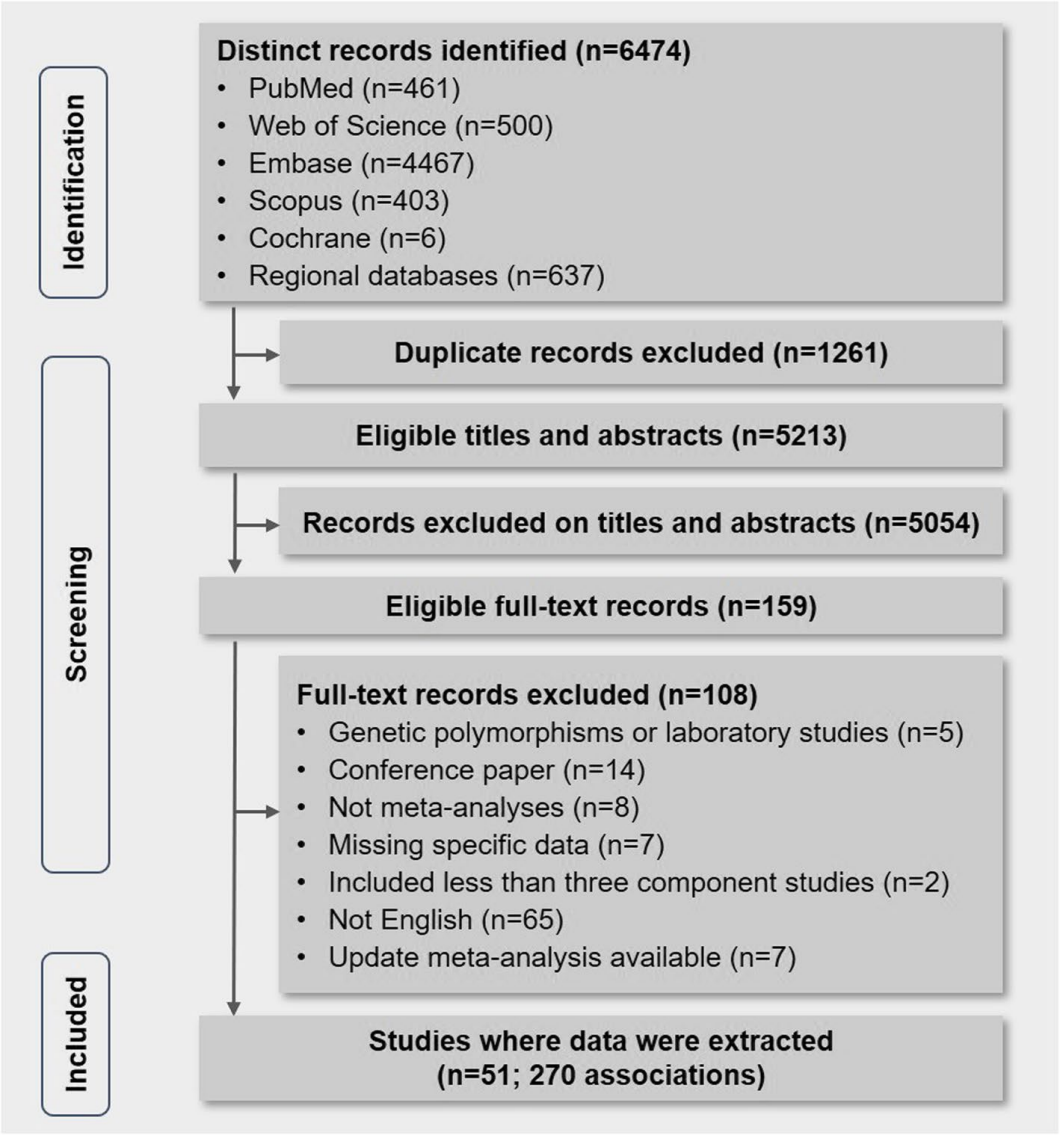
PRISMA flow chart. Flow chart of included and excluded systematic reviews and meta-analyses

We retrieved 6474 records from PubMed, Web of Science, Embase, Scopus, Cochrane Library, and several regional databases. According to the criterion, 159 full-text articles were retrieved and checked for inclusion after duplicate removal, title, and abstract screening. There were no additional eligible articles found by hand-checking the reference lists of all systematic reviews. Overall, 51 systematic reviews with meta-analyses corresponded to 270 unique associations were included [7, 9, 11, 12, 31–33, 35–78] (Fig. 1). The two pairs of four investigators showed high consistency in terms of study selection, with kappa values of 0.893 and 0.926, respectively. The excluded articles and the reasons behind their removal are provided in Additional file 1: Table S5. For meta-analyses excluded due to a lack of data relating to quantitative synthesis, we further summarized their findings in Additional file 1: Table S6.

### Characteristics of included meta-analyses

**Fig. 2:**
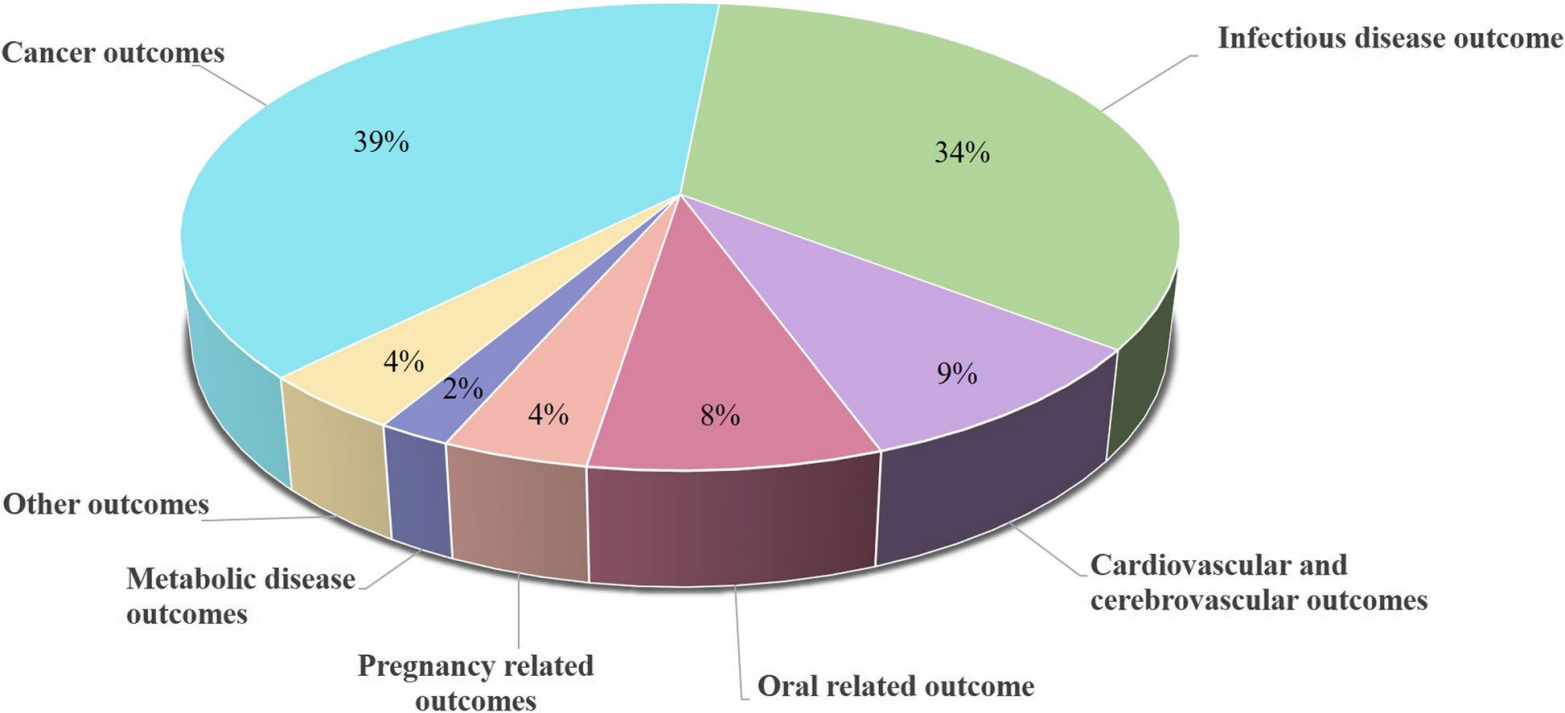
Map of 270 blood group related outcomes: percentage of outcomes per outcome category for all studies

The 51 systematic reviews with meta-analyses corresponded to 270 unique associations: 105 on cancer outcomes (39%), 91 on infectious outcomes (34%), 25 on cardiovascular outcomes (9%), 22 on oral-related outcomes (8%), 12 on pregnancy-related outcomes (4%), 5 on metabolic disease (2%), and 10 on other outcomes (4%) (Fig. 2). The systematic reviews with meta-analyses included in this UR were published from 2007 until 2023. The number of studies per association ranged from 3 to 49. One hundred and ninety-five meta-analyses included ≥  1000 cases (Additional file 1: Table S7).

### Summary findings

Among 270 associations included in our UR. Eighty-nine associations (33%) presented a nominally statistically significant effect (*P* < 0.05). Of these, 41 (46%) were in conformity with the principle of statistical significance at *P* < 10^ −3^, and 24 (27%) reached *P* < 10^ −6^. When calculating the effect size of the largest data study of the associations, 61 (69%) of the 89 associations showed statistical significance. After estimating the 95% PI, 66 (74%) contained null values. Twenty-three (26%) and 19 (21%) associations had significant (50% < *I*^2^ ≤ 75%) and considerable (*I*^2^ > 75%) heterogeneity estimates, respectively. Twenty-one (24%) of the 89 associations presented evidence for small-study effects, and 29 (33%) associations presented evidence for excess significance bias.

### Cancer outcomes

**Fig. 3:**
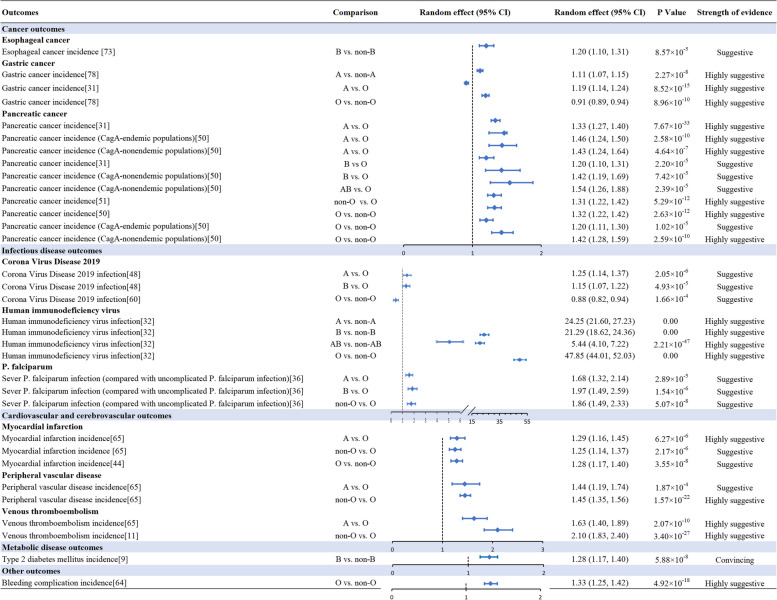
Forest plot showing studies investigating the association between blood group and health outcomes. CI, confidence interval; CagA, cytotoxin-associated gene A

We summarized 105 associations between blood group and cancer outcomes. The magnitude of the observed summary random effects estimates ranged from 0.65 to 1.54 (Additional file 2: Fig. S1). Thirty-one meta-analyses (30%) presented a nominally statistically significant effect (*P* < 0.05). Of these, 14 associations were graded as suggestive or above evidence (Fig. 3, Additional file 1: Tables S7–8).

#### Esophageal cancer

We found blood group B was associated with a higher risk of esophageal cancer (OR =  1.20; 95% CI: 1.10, 1.31), compared with blood group non-B. And the association was graded as suggestive evidence.

#### Gastric cancer

Blood group A was associated with a higher risk of gastric cancer, both compared with blood group non-A (OR =  1.11; 95% CI: 1.07, 1.15) and blood group O (OR =  1.19; 95% CI: 1.14, 1.24). However, blood group O was associated with a lower risk of gastric cancer (OR =  0.91; 95% CI: 0.89, 0.94), compared with blood group non-O. These above associations were graded as highly suggestive evidence.

#### Pancreatic cancer

Compared with blood group O, blood group A was associated with higher risk of pancreatic cancer (OR =  1.33; 95% CI: 1.27, 1.40), cytotoxin-associated gene A (CagA) endemic pancreatic cancer (OR =  1.46; 95% CI: 1.24, 1.50) and CagA-nonendemic pancreatic cancer (OR =  1.43; 95% CI: 1.24, 1.64); blood group B was associated with higher risk of pancreatic cancer (OR =  1.20; 95% CI: 1.10, 1.31) and CagA-nonendemic pancreatic cancer (OR =  1.42; 95% CI: 1.19, 1.69); blood group AB was associated with higher risk of CagA-nonendemic pancreatic cancer (OR =  1.54; 95% CI: 1.26, 1.88); Blood group non-O was also associated with higher risk of pancreatic cancer (OR =  1.31; 95% CI: 1.22, 1.42). Compared with blood group non-O, blood group O was associated with a higher risk of pancreatic cancer (OR =  1.32; 95% CI: 1.22, 1.42), CagA endemic pancreatic cancer (OR =  1.20; 95% CI: 1.11, 1.30), and CagA-nonendemic pancreatic cancer (OR =  1.42; 95% CI: 1.28, 1.59). According to the UR criteria, the associations between blood group A and pancreatic cancer, CagA endemic, and CagA-nonendemic pancreatic cancer risk, blood group O and pancreatic cancer and CagA-nonendemic pancreatic cancer, blood group non-O and pancreatic cancer were graded as highly suggestive evidence. The remaining associations were graded as suggestive evidence.

### Infectious disease outcomes

Ninety-one associations between blood group and infectious disease outcomes were investigated. The magnitude of the observed summary random effects estimates ranged from 0.50 to 47.85 (Additional file 2: Fig. S2). Overall, 27 (32%) of 85 associations reached a statistically significant value at *P* < 0.05. Ten associations were supported by suggestive or above evidence (Fig. 3, Additional file 1: Tables S7–8).

#### Coronavirus disease 2019 (COVID-19)

We found blood group A (OR =  1.25; 95% CI: 1.14, 1.37) and blood group B (OR =  1.15; 95% CI: 1.07, 1.22) were associated with an increased risk of COVID-19 infection, compared with blood group O. But blood group O was associated with a decreased risk of COVID-19 infection (OR =  0.88; 95% CI: 0.82, 0.94), compared with blood group O. The association between blood group O and COVID-19 infection was supported by highly suggestive evidence. The other two associations were supported by suggestive evidence.

#### Human immunodeficiency virus (HIV)

Four blood groups of ABO blood group system were associated with an increased risk of HIV infection (RR =  24.25; 95% CI: 21.60, 27.23; blood group A versus blood group non-A, RR =  21.29; 95% CI: 18.62, 24.36; blood group B versus blood group non-B, RR =  5.44; 95% CI: 4.10, 7.22; blood group AB versus blood group non-AB, and RR =  47.85; 95% CI: 44.01, 52.03; blood group O versus blood group non-O). And these four associations were supported by highly suggestive evidence.

#### *P. falciparum*

Blood group A (OR =  1.68; 95% CI:1.32, 2.14), blood group B (OR =  1.97; 95% CI:1.49, 2.59), and blood group non-O (OR =  1.86; 95% CI:1.49, 2.33) were associated with an increased risk of *P. falciparum* infection, compared with blood group O. All of these associations were supported by suggestive evidence.

### Cardiovascular and cerebrovascular outcomes

Twenty-five associations between blood group and cardiovascular and cerebrovascular outcomes were summarized. The magnitude of the observed summary random effects estimates ranged from 0.58 to 2.55 (Additional file 2: Fig. S3). Of which, 21 (84%) associations gave a show on statistically significant effect nominally (*P* < 0.05), and 7 associations reached suggestive or above evidence (Fig. 3, Additional file 1: Tables S7–8).

#### Myocardial infarction (MI)

Blood group A (OR =  1.29; 95% CI: 1.16, 1.45) and blood group non-O (OR =  1.25; 95% CI:1.14, 1.37) had an increased risk of MI compared with blood group O. Another association showed blood group O had an increased risk of MI (OR =  1.28; 95% CI:1.17, 1.40) compared with blood group non-O. All three associations reached suggestive evidence.

#### Peripheral vascular disease (PVD)

Compared with blood group O, blood group A (OR =  1.44; 95% CI: 1.19, 1.74) and blood group non-O (OR =  1.45; 95% CI:1.35, 1.56) had an increased risk of PVD. The associations between blood group A and blood group non-O and PVD risk were reached suggestive and highly suggestive evidence, respectively.

#### Venous thromboembolism (VTE)

Blood group A (OR =  1.63; 95% CI: 1.40, 1.89) and blood group non-O (OR =  2.10; 95% CI:1.83, 2.40) had an increased risk of VTE compared with blood group O. And the two associations reached highly suggestive evidence.

### Oral-related outcome

Twenty-two associations between blood group and oral-related outcomes were summarized. The magnitude of the observed summary random effects estimates ranged from 0.70 to 1.36 (Additional file 2: Fig. S4). Only one association gave a show on statistically significant effect nominally (*P* < 0.05). No association reached suggestive or above evidence (Additional file 1: Tables S7–8).

### Pregnancy-related outcomes

We summarized twelve associations between blood group and pregnancy-related outcomes. The summary random effects estimate magnitude ranged from 0.90 to 1.49 (Additional file 2: Fig. S5). Only two associations were statistically significant at *P* < 0.05. And no association reached suggestive or above evidence (Additional file 1: Tables S7–8).

### Metabolic disease outcomes

We summarized 5 associations between blood group and metabolic disease outcomes. The magnitude of the observed summary random effects estimates ranged from 0.91 to 1.28 (Additional file 2: Fig. S6). Only 2 (40%) of 5 associations were nominally statistically significant at a *P* < 0.05 level. One association was supported by suggestive or above evidence (Fig. 3, Additional file 1: Tables S7–8).

#### Type 2 diabetes mellitus incidence (T2DM)

Blood group B, compared with blood group non-B, had a greater risk of T2DM (OR =  1.28; 95% CI: 1.17, 1.40), and the association was supported by convincing evidence.

### Other outcomes

Ten associations between blood group and other outcomes (such as bleeding complication, decreased ovarian reserve) were summarized. The summary random effects estimate magnitude ranged from 0.84 to 1.33 (Additional file 2: Fig. S7). Only one association was statistically significant at *P* < 0.05. And which was supported by highly suggestive evidence (Fig. 3, Additional file 1: Tables S7–8).

#### Bleeding complication

Blood group O was associated with a higher risk of bleeding complication (OR =  1.33; 95% CI: 1.25, 1.42), compared with blood group non-O, which was supported by highly suggestive evidence.

In summary, we found an association between blood group B and an increased risk of T2DM incidence (OR =  1.28; 95% CI: 1.17, 1.40) was rated as convincing evidence when it was taken as comparison for blood group non-B, by owing over 1000 cases, random *P* value < 10^–6^, not large heterogeneity (*I*^2^ < 50%), 95% PI excluding the null value, no hints for small-study effects and excess significance bias (Fig. 3). Eighteen associations were rated as highly suggestive evidence, they reached a statistically significant value at *P* < 10^ −6^, had more 1000 cases, and the *P* value of the largest study was less than 0.05, such as comparison with blood group O, both blood group A (OR =  1.63; 95% CI: 1.40, 1.89) and non-O blood group (OR =  2.10; 95% CI: 1.83, 2.40) increased the risk of VTE incidence. In addition, we found 14 associations were rated as suggestive evidence. Fifty-six associations were rated as weak evidence and the remaining 181 associations were not significant (Additional file 1: Tables S7–8).

### Methodological quality of the meta-analyses

With the measurement tool AMSTAR, 18 (35%) articles were categorized as high quality. Of the 51 articles, 32 (63%) articles and only 1 (2%) article were categorized as moderate and low quality, respectively (Additional file 1: Table S9).

### Certainty of the evidence

Based on the GRADE approach, no health outcomes reached high credibility criteria. Nine of 89 health outcomes met the moderate certainty criteria. Thirty-three and 47 of 89 health outcomes met the low and very low certainty criteria, respectively (Additional file 1: Table S10).

### Sensitivity analyses

Findings from sensitivity analyses are reported in Additional file 1: Tables S11–13. Removal of small-sized studies from the meta-analyses with evidence of small-study effects, these evidence ratings were not modified. When excluding low-quality studies, the associations between blood group A and gastric cancer, pancreatic cancer, and VTE and blood group O and pancreatic cancer retained their highly suggestive evidence ratings. When we focused on the associations excluded due to overlap, twelve associations were downgraded because of random *P* value.

## Discussion

### Main findings

This is the first UR to provide a comprehensive overview of the observational data assessing associations between the ABO and Rh blood groups and multiple health outcomes. And we found 89 statistically significant associations. Convincing (Class I) evidence was only presented for the association between blood group B and T2DM risk. Highly suggestive (Class II) evidence was presented for 18 associations, such as HIV and VTE.

### Comparison with previous studies

The positive association between blood group B and the risk of T2DM detected in this UR was supported by a prospective cohort study. This study included 82,104 women and followed for 18 years in France, throughout which 3553 women had a validated diagnosis of T2DM. After adjustment for potential confounders, blood group B increased the risk of T2DM compared with blood group O (HR =  1.21; 95% CI: 1.07, 1.36) [79]. A comparative cross-sectional study, including 326 participants (163 T2DM patients and 163 age and sex-matched healthy individuals), confirmed the harmful association of blood group B with T2DM risks (OR =  1.96; 95% CI: 1.05, 3.65), compared with the non-B blood group [80]. A meta-analysis revealed blood group B was significantly associated with an increased risk of T2DM (RR =  1.05; 95% CI: 0.93, 1.18), compared with the non-B blood group [81]. Nevertheless, caution is warranted in interpreting the observed association between blood group B and T2DM risk. Despite our result being consistent with findings from a prospective cohort study conducted in France, it is important to note that they exclusively included women. Subgroup analysis stratified by gender is needed in the future. In addition, the above studies have different control groups, sample sizes, and study designs. Further well-designed, large-scale prospective studies are needed to clarify the association between blood group B and T2DM.

The association between blood groups and HIV infection wase debated. In our UR, we found all ABO blood group was associated with an increased risk of HIV infection, and all of them were supported by highly suggestive evidence. A cross-sectional study conducted in Nairobi, Kenya among 280 female sex workers showed blood group A (OR =  1.56; 95% CI: 1.06, 2.28) was associated with HIV infection, compared with blood group O. However, blood group B (OR =  1.63; 95% CI: 0.94, 2.80) and blood group AB (OR =  1.50; 95% CI: 0.57, 3.93) were not associated with HIV infection [82]. A previous cross-sectional study conducted in South Africa investigated the associations between ABO blood groups and HIV infection among blood donors. The results suggested that the ABO blood group was not related to HIV infection. However, the point estimate for OR assesses blood group AB and HIV infection is 1.03 [83]. Cross-sectional studies cannot be used to infer causality and potential biases should be considered in the observational studies. Further well-designed longitudinal studies and controlling for different sources of bias are warranted to assess causality.

The harmful association between blood group A and non-O blood group and VTE incidence observed in our UR was supported by previous studies. For example, a previous study that included 7830 patients found blood group A was associated with VTE incidence (OR =  2.16; 95% CI: 1.10, 4.24) [84] in comparison with the blood group.

### Biological plausibility

Multifactorial mechanisms might explain the increased risk of T2DM associated with blood groups. The previous study showed ABO blood group is in association with the level of plasma soluble intercellular adhesion molecule-1 and tumor necrosis factor receptor-2 [85]. And the above markers are identified to contribute a higher risk of T2DM. Moreover, a study suggested that the ABO blood group, being a gene-determined host factor, modulated the composition of the intestinal microbiota [86], which played an important role in influencing metabolism including glucose metabolism, energy balance, and low-grade inflammation [87].

For potential mechanisms between blood group and HIV infection, some studies indicated that expression of glycosyltransferase could be induced due to HIV and further synthetization of antigens of blood type on lymphocyte surfaces [88, 89]. Therefore, apart from releasing new virion particles from lymphocytes, HIV could also integrate antigens of the blood group into its envelope surface [89]. The presence of these antigens sensitizes the virus against neutralizing antibodies and complements specific blood groups, potentially influencing the virus’s transmission between individuals and different blood groups [88].

It has not been thoroughly clear of the exact mechanism revealing the ABO blood group and VTE. The most likely hypothesis is that ABO plays a role in dominating the glycosylation degree of von Willebrand factor via modifying GT expressions [90]. von Willebrand factor multimeric composition is regulated in plasma by ADAMTS13. Proteolysis is enhanced by von Willebrand factor deglycosylation by ADAMTS13 [91]. In addition, individuals with blood group A1 and blood group B are at the level of 20% higher circulating Willebrand factor on average, which factor VIII levels than for O or A2 [92, 93], high plasma levels of Willebrand factor, and factor VIII having association with increased VTE risk [94–97].

### Strengths and limitations

To our knowledge, this is the first UR that systematically and comprehensively appraises the hierarchy of evidence relating blood groups to various health outcomes. Beyond summarizing the findings for a series of health end-point, we further an inquiry into bias and heterogeneity in the observational blood group literature. Compared with an individual systematic review or meta-analysis. This UR helped to summarize the complicated and vast amounts of research by comparing and contrasting the results of individual reviews, which provided an efficient overview of the findings for a particular problem [98]. Moreover, we adhered to a systematic methodology involving a search strategy in electronic databases and study selection and extraction conducted by two separate researchers. We also used standard approaches to evaluate the methodological quality and epidemiological evidence strength of the included studies.

UR provides top-tier evidence and important insights, but several limitations should be considered. First, some systematic reviews and meta-analyses did not acquire the level of evidence because they did not provide the number of cases. Second, we used *I*^2^ (an estimate of the proportion of variance reflecting true differences in effect size) and *τ*^2^ (an estimate of true variation in the summary estimate) to evaluate statistical heterogeneity. According to UR criteria, *I*^2^ < 50% was applied as one of the criteria for convincing evidence in our UR, assigning the best evidence grade to robust associations. Several systematic reviews with meta-analyses examined the clinical and methodological heterogeneity by performing subgroup analyses stratified by these characteristics. Of note, we also extracted this information and analyzed it in the present UR. For example, within the subgroup comprising pancreatic cancer patients classified as either CagA-nonendemic or CagA endemic and COVID-19 patients with hospitalization, we found the results from subgroup analyses were consistent with the main findings. Future studies should better explore clinical and methodological heterogeneity to verify the association between blood groups and various health outcomes. Third, for the same health outcome (e.g., COVID-19 infection), the comparison group is different (e.g., A vs B, A vs AB, A vs O, and A vs non-A). Therefore, the findings between the blood group and health outcome in our study should be interpreted with caution. Fourth, we identified studies from published systematic reviews with meta-analyses, which may have omitted some individual studies for not in the systematic reviews with meta-analyses above. However, the systematic reviews with meta-analyses included in the current study were those of included the largest number of primary studies, which was unlikely to affect our results. Fifth, the reliability of the UR relies directly on the incorporated systematic reviews with meta-analyses. However, some included systematic reviews with meta-analyses existed risk of bias, which might decrease the robustness of statistical analyses. The study did not adjust for confounding factors that could have mediated associations between blood group and outcomes, because adjustment for potential confounders was unavailable in published systematic reviews with meta-analyses. Sixth, as this UR only included observational data, limitations common to this approach may influence the results of this review, such as information bias and residual confounding. There was a limited number of systematic reviews with meta-analyses that exclusively included prospective study designs, where information bias was reduced. However, case–control and cross-sectional study designs were more common than prospective study designs and were associated with a higher potential for information bias and reverse causation.

## Conclusions

This comprehensive UR will help investigators to judge the relative priority of health outcomes related to the ABO blood group and RH blood group for future research and clinical management of the disease. In summary, compared with the non-B blood group, we found the association between blood group B and increased risk of T2DM incidence (OR =  1.28; 95% CI: 1.17, 1.40) was supported by convincing evidence. We also found 18 associations, such as blood group A and the risk of VTE incidence (OR =  1.63; 95% CI: 1.40, 1.89) and non-O blood group and the risk of VTE incidence (OR =  2.10; 95% CI: 1.83, 2.40), were supported by highly suggestive evidence. To enhance the quality of evidence regarding these associations and be able to give strong recommendations, future studies should consider several aspects. For example, set the same control group to increase the comparability of results, use standard definition of exposure or outcome to reduce clinical heterogeneity, and match the characteristics between cases and controls to reduce the impact of potential confounding. In addition, future studies understanding mechanisms between blood groups and various health outcomes are needed.

### Supplementary Information

Additional file 1: Tables S1-13. Table S1-PRISMA checklist of items to include when reporting a systematic review or meta-analysis; Table S2-MOOSE checklist for meta-analyses of observational studies; Table S3-Search strategy; Table S4-Criteria for categorizing the credibility of evidence in the umbrella review; Table S5-The list of the excluded records during the process of full-text review; Table S6-The summary results of meta-analyses excluded due to lack of data for quantitative synthesis; Table S7-Description of 270 associations investigating the associations between ABO and Rhesus blood groups and multiple health outcomes; Table S8-Strength assessment of evidence from 270 associations examining associations between ABO and Rhesus blood groups and multiple health outcomes; Table S9-Methodological quality assessment of the included articles with AMSTAR; Table S10-The results of GRADE assessment of the evidence certainty on the associations between ABO and Rhesus blood groups and multiple health outcomes; Table S11-Sensitivity analysis results of omission of small-sized studies (< 25th percentile) from those meta-analyses with evidence of small-study effects; Table S12-Sensitivity analysis results of omission of primary studies with low-quality evidence; Table S13-Sensitivity analysis results of excluded meta-analyses due to overlap.

Additional file 2: Fig. S1-7. Fig. S1-Summary effects sizes with inverse of the variance of association between blood group and cancer outcomes; Fig. S2-Summary effects sizes with inverse of the variance of association between blood group and infectious disease outcomes; Fig. S3-Summary effects sizes with inverse of the variance of association between blood group and cardiovascular and cerebrovascular outcomes; Fig. S4-Summary effects sizes with inverse of the variance of association between blood group and oral related outcomes; Fig. S5-Summary effects sizes with inverse of the variance of association between blood group and pregnancy related outcomes; Fig. S6-Summary effects sizes with inverse of the variance of association between blood group and metabolic disease outcomes; Fig. S7-Summary effects sizes with inverse of the variance of association between blood group and other outcomes.

## Data Availability

Not applicable.

## Abbreviations

AMSTAR: A Measurement Tool to Assess Systematic Reviews
CIs: Confidence intervals
HIV: Human immunodeficiency virus infection
HR: Hazard ratio
OR: Odds ratio
PIs: Prediction intervals
PVD: Peripheral vascular disease
Rh: Rhesus
RR: Risk ratio
T2DM: Type 2 diabetes mellitus
UR: Umbrella review
VTE: Venous thromboembolism
