# Correction: ABO and Rhesus blood groups and multiple health outcomes: an umbrella review of systematic reviews with meta-analyses of observational studies

**DOI:** 10.1186/s12916-024-03520-x

**Published:** 2024-07-11

**Authors:** Fang-Hua Liu, Jia-Kai Guo, Wei-Yi Xing, Xue-Li Bai, Yu-Jiao Chang, Zhao Lu, Miao Yang, Ying Yang, Wen-Jing Li, Xian-Xian Jia, Tao Zhang, Jing Yang, Jun-Tong Chen, Song Gao, Lang Wu, De-Yu Zhang, Chuan Liu, Ting-Ting Gong, Qi-Jun Wu

**Affiliations:** 1 Department of Clinical Epidemiology, Shengjing Hospital of China Medical University, Shenyang, China; 2 Clinical Research Center, Shengjing Hospital of China Medical University, Shenyang, China; 3 Hospital Management Office, Shengjing Hospital of China Medical University, Shenyang, China; 4 Department of Obstetrics and Gynecology, The Fourth Affiliated Hospital of China Medical University, Shenyang, China; 5 Department of Radiology, Shengjing Hospital of China Medical University, Shenyang, China; 6 Department of Pediatrics, Shengjing Hospital of China Medical University, Shenyang, China; 7 Department of Hematology, Shengjing Hospital of China Medical University, Shenyang, China; 8 Department of Otolaryngology Head and Neck Surgery, Shengjing Hospital of China Medical University, Shenyang, China; 9 Department of Endocrinology, Shengjing Hospital of China Medical University, Shenyang, China; 10 School of Medicine, Zhejiang University, Hangzhou, China; 11 Cancer Epidemiology Division, Population Sciences in the Pacific Program, University of Hawaii Cancer Center, University of Hawaii at Manoa, Honolulu, HI USA; 12 NHC Key Laboratory of Advanced Reproductive Medicine and Fertility (China Medical University), National Health Commission, Shenyang, China; 13 Department of Obstetrics and Gynecology, Shengjing Hospital of China Medical University, Shenyang, China

**Correction** **:** **BMC Med 22, 206 (2024)**

**https://doi.org/10.1186/s12916-024-03423-x**

In the original article [1], five co-authors - Song Gao, De-Yu Zhang, Chuan Liu, Ting-Ting Gong, and Qi-Jun Wu - are all erroneously affiliated to affiliation #4 in the original article ("Department of Obstetrics and Gynecology, The Fourth Affiliated Hospital of China Medical University, Shenyang, China").

Affiliation #4 for each of the above-named authors should instead replaced by a new affiliation #13 with the following details as also demonstrated in the authorship of this correction article: "Department of Obstetrics and Gynecology, Shengjing Hospital of China Medical University, Shenyang, China"

Affiliation #4 itself remains correct and affiliated solely to co-author, Xue-Li Bai.
