# Supplementary material for: ABO and Rhesus blood groups and multiple health outcomes: an umbrella review of systematic reviews with meta-analyses of observational studies
Source: BMC Med. 2024 May 20;22:206. doi: 10.1186/s12916-024-03423-x (PMC11106863; doi:10.1186/s12916-024-03423-x)
Supplement: Supplementary file 2 — Additional file 2: Fig. S1-7. Fig. S1-Summary effects sizes with inverse of the variance of association between blood group and cancer outcomes; Fig. S2-Summary effects sizes with inverse of the variance of association between blood group and infectious disease outcomes; Fig. S3-Summary effects sizes with inverse of the variance of association between blood group and cardiovascular and cerebrovascular outcomes; Fig. S4-Summary effects sizes with inverse of the variance of association between blood group and oral related outcomes; Fig. S5-Summary effects sizes with inverse of the variance of association between blood group and pregnancy related outcomes; Fig. S6-Summary effects sizes with inverse of the variance of association between blood group and metabolic disease outcomes; Fig. S7-Summary effects sizes with inverse of the variance of association between blood group and other outcomes. [file 12916_2024_3423_MOESM2_ESM.docx]

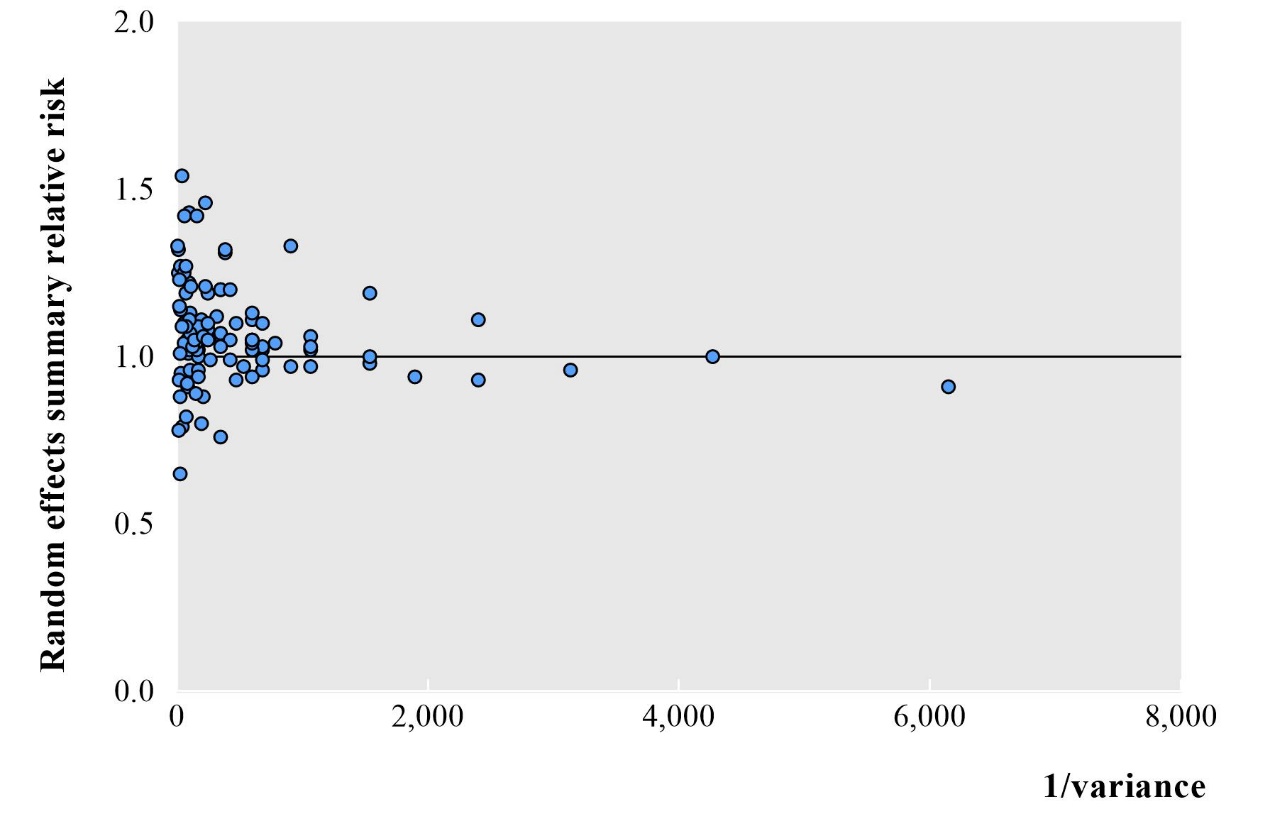


**Fig. S1-Summary effects sizes with inverse of the variance** **of** **association between blood group and cancer outcomes**


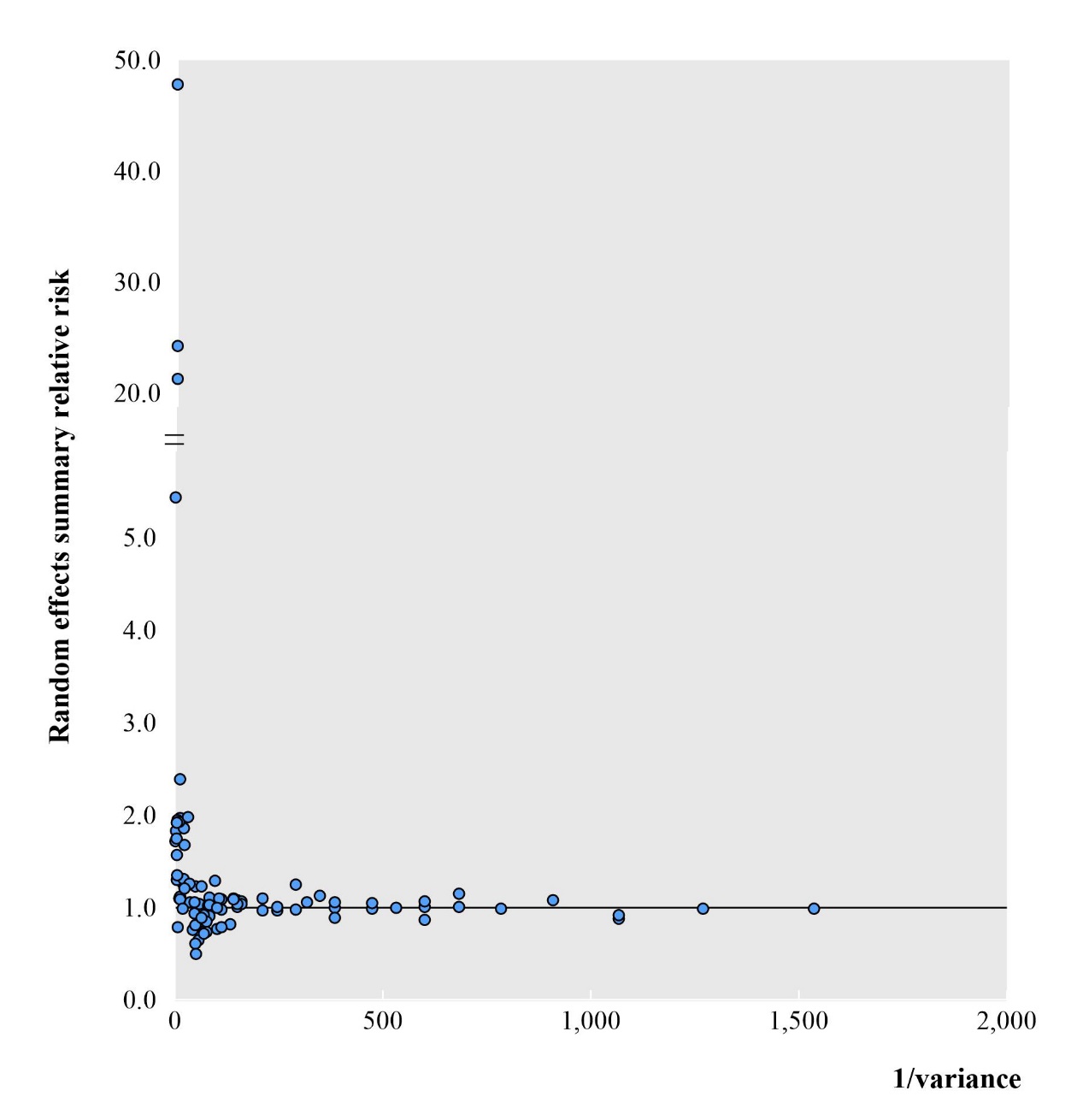


**Fig. S2-Summary effects sizes with inverse of the variance** **of** **association between blood group and infectious disease outcomes**


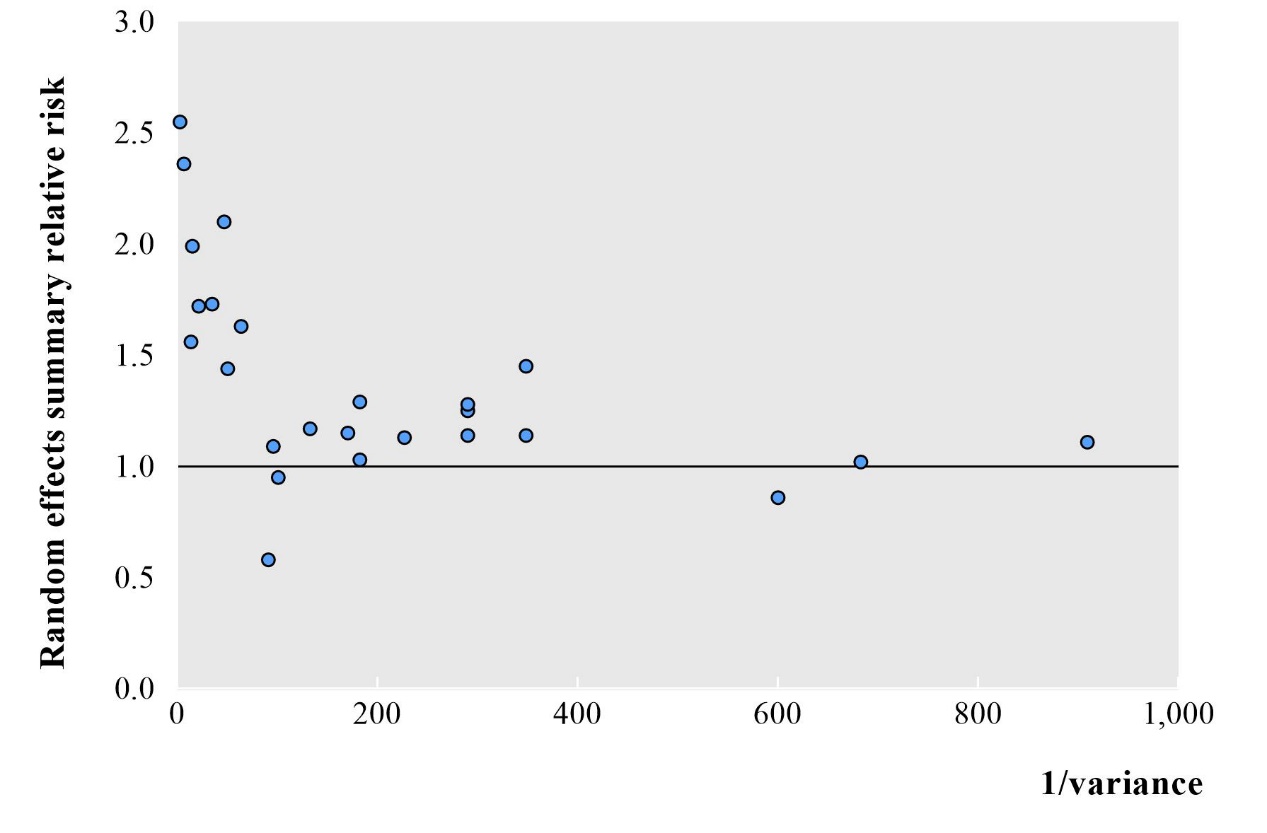


**Fig. S3-Summary effects sizes with inverse of the variance** **of** **association between blood group and cardiovascular and cerebrovascular outcomes**


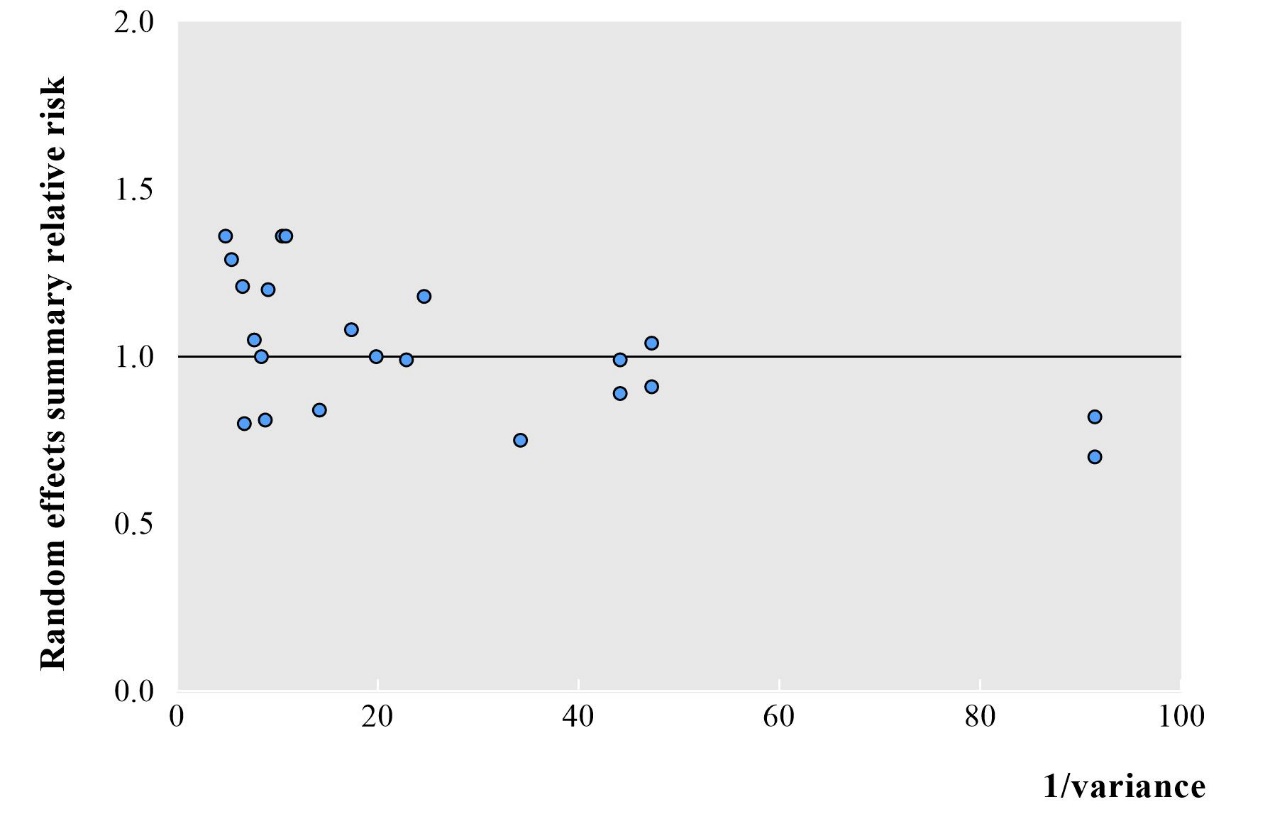


**Fig. S4-Summary effects sizes with inverse of the variance** **of** **association between blood group and oral related outcomes**

**Fig. S5-Summary effects sizes with inverse of the variance** **of** **association between blood group and pregnancy related outcomes**

**Fig. S6-Summary effects sizes with inverse of the variance** **of** **association between blood group and metabolic disease outcomes**


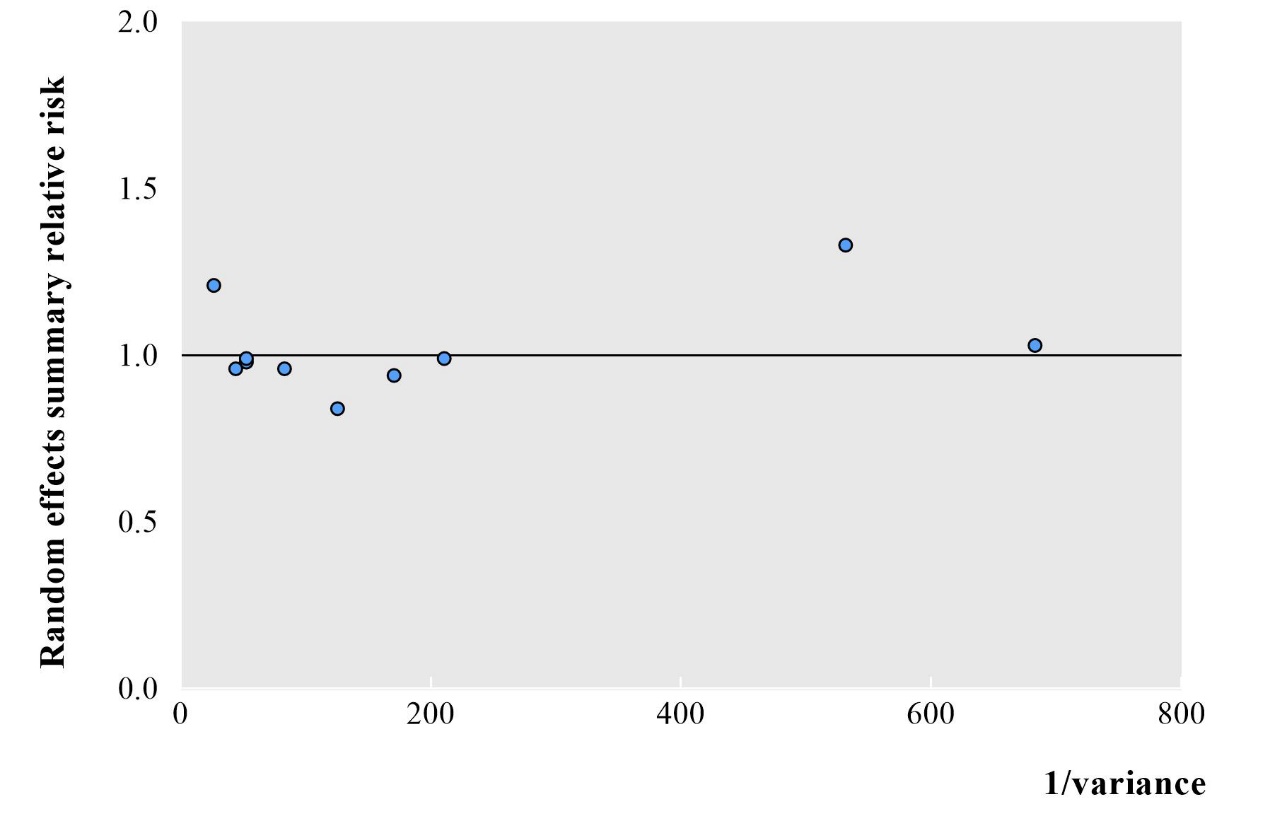


**Fig. S7-Summary effects sizes with inverse of the variance** **of** **association between blood group and other outcomes**
